# Detection of *Escherichia coli* O157:H7 in imported meat products from Saudi Arabian ports in 2017

**DOI:** 10.1038/s41598-023-30486-2

**Published:** 2023-03-14

**Authors:** Meshari Ahmed Alhadlaq, Mohammed I. Mujallad, Sulaiman M. I. Alajel

**Affiliations:** Section of Molecular Biology, Reference Laboratory for Microbiology, Research & Laboratories Sector, Saudi Food and Drug Authority, Abdulaziz Bin Abdullah Street, Sina’iyah District, Riyadh, 12843 Saudi Arabia

**Keywords:** Epidemiology, Microbiology, Molecular biology, Health care

## Abstract

*Escherichia coli* O157:H7 is a foodborne pathogen, which causes various health conditions in humans, including fatigue, nausea, bloody diarrhoea and in some cases, even death. In 2017, 15.71% of the total imported food products in Saudi Arabia (SA) were meat-based. India and Brazil are two of the top five countries from where SA imports meat. According to the Saudi Food and Drug Authority, in 2017, at least 562, 280, and 50 samples of imported beef, chicken and sheep meat, respectively, were tested for the presence of *E. coli* O157:H7. Amongst these, *E. coli* O157:H7 was detected in respectively 6.80% and 2.20% of the tested beef meat samples imported from India and Brazil as well as in respectively 6.96% and 3.57% of the tested chicken samples imported from Brazil and Ukraine. Moreover, the pathogen was detected in 2.13% of the tested sheep meat samples imported from India. The present report provides evidence that imported meat can serve as the carrier of *E. coli* O157:H7, which may lead to epidemics within the Kingdom of Saudi Arabia.

## Introduction

*Escherichia coli* is a Gram-negative, facultative, anaerobic bacterium considered to be a commensal organism in the human body^1^. However, the *E. coli* strain O157:H7 is a pathogen that poses a threat to human life by causing several diseases, such as haemolytic–uraemic syndrome (HUS), which may be fatal in some cases^2^. The primary reservoir of *E. coli* O157:H7 is meat, although it has also been isolated from fruits and vegetables^3,4^. The O157:H7 strain was first detected in 1982. Within only two decades (1982–2002), it has been responsible for 73,000 illnesses annually in the United States alone, causing as many as 350 outbreaks^5^. Illnesses caused by *E. coli* O157:H7 have been reported in over 30 countries across six continents^6^.

*Escherichia coli* strains that produce Shiga toxins (Stx1 and Stx2) are called Shigatoxigenic *E. coli* (STEC)^7^, while those that produce Shiga-like toxins (verotoxins) are called verotoxigenic *E. coli* (VTEC)^8^. The pathogenicity of STEC is associated with virulence factors such as enterohaemolysin (encoded by *hlyA*), intimin (encoded by *eae*) and Stx1 and Stx2 (encoded by *stx1* and *stx2*)^7^. STEC isolates are further divided into two groups: O157 and non-O157^1^. O157 isolates belong to the H7 and NM serogroups, whereas non-O157 isolates belong to the O26, O45, O103, O111, O121, and O145 serogroups^1,5^. Notably, O157, O26, O103, O111 and O145 are also classified as enterohaemorrhagic *E. coli* (EHEC)^9^. Interestingly, a comprehensive *E. coli* O157:H7 clade-typing study (clades 1–9) of 269 HUS patients and 387 asymptomatic carriers (ACs) in Japan between 1999 and 2011 reported that clades 6 and 8 were frequently found in HUS patients^10^. Furthermore, the *norV* gene, which codes a nitric oxide reductase (Shiga toxin inhibitor in anaerobic conditions), was found intact in clade 1–3 isolates but not in clade 4–8 isolates^10^.

In Saudi Arabia (SA), no *E. coli* O157:H7 outbreak has been reported to date, and the prevalence of this pathogen remains unknown. However, it has been isolated from several local cattle farms^11^. Reporting outbreaks in SA is challenging because of its inefficient data collection system^12^. For this reason, since 2003, the Saudi Food and Drug Authority (SFDA) has taken control of all food safety regulations, which has also helped avoid overlapping with other authorities^13^. As a member of the Gulf Cooperation Council (GCC), SA is required to apply the GCC Standardization Organization’s (GSO) microbiological criteria for foodstuffs [GSO/1016/2015 (E)] E: referring to the English version^14^. Accordingly, the SFDA labs follow the GSO 2015 guideline stating that all kinds of food must be free from *E. coli* O157:H7.

Statistical information on food imported into SA over the past decade is limited. A recent study identified the main source of imported meat only in 2017^13^. Approximately 80% of the food available in Saudi Arabian markets is imported, and 15.71% of it is meat-based^13^. Therefore, the main aim of this study was to compare imported meat contaminated with *E. coli* O157:H7 with the total meat imported in 2017. To that end, the study evaluated the possibility of detecting *E. coli* O157:H7 in meat products imported into SA in 2017 using the SFDA’s monitoring system to provide foundational data for creating a database of the O157:H7 serotype.

## Methods

### Sample collection

The data used in this study were extracted from the laboratory information management system (LIMS) of the SFDA database, an online tool for data management operated by LabVantage Solutions, Inc. Typically, when shipments of imported consumable meat arrive at Saudi port customs, SFDA inspectors collect samples and send them to SFDA labs for analysis. Thereafter, the inspected samples are referred for *E. coli* O157:H7 detection. The data used in this study pertained to analyses of raw (not ready-to-eat ‘RTE’) products only. Sample’s specific details can be found in Supplementary Table 1, 2, 3 and 4.

### *E. coli* O157:H7 detection

#### Enrichment

Samples weighing 25 g selected for enrichment were placed in sterilised sample bags. They were then homogenised with 225 mL of modified tryptone soya broth (mTSB) supplemented with novobiocin to obtain a ratio as follows: mTSB + sample of 1/10 (mass to volume). The sample bags were massaged by hand and then incubated at 41.5 °C for 12–18 h. *Escherichia coli* O157 strain ATCC 43895 and blank were added as positive and negative controls, respectively. After incubation, the samples were subjected to immunomagnetic separation. Subsequently, 50 µL of each sample was streaked out on pre-dried cefixime tellurite sorbitol MacConkey (CT-SMAC) agar plates using sterile loops to obtain many well-isolated colonies and incubated at 37 °C for 18–24 h.

#### Colony selection

After incubation, at least five presumptive colonies were selected randomly from each plate and placed into polymerase chain reaction (PCR) tubes containing 10 µL of distilled water (dH_2_O) as a preparation step for DNA extraction.

#### DNA extraction

The samples were prepared using a PrepMan™ Ultra Sample Preparation Reagent Kit (lot number 1809191) according to the manufacturer’s protocol.

#### PCR detection

Real-time PCR (RT-PCR) was performed to amplify the O157:H7-specific target DNA sequences using a MicroSEQ™ *E. coli* O157:H7 Detection Kit (lot number 1804034) according to the manufacturer’s protocol. Non-pathogenic *E. coli* ATCC 25922, non-O157 'O111 and O26' and *Salmonella* strains *enteritis* and *arizona* were added as negative controls. A 7500 Fast System and Sequence Detection System (SDS) software v1.4.2 were used for the analysis. Each sample was analysed in triplicate. The thermal cycling conditions are displayed in Supplementary Table 5. International Organization for Standardization (ISO) 17025 (2017) and 13136 (2012) were used in SFDA labs and to isolate *E. coli* O157:H7, respectively^15,16^.

### Statistical analysis

Statistical analyses were performed using Microsoft Office Excel Professional Plus 2019. For pairwise comparisons, the *t*-test was used to compare between samples to assess differences in the prevalence of *E. coli* O157:H7. Values of *P* < 0.05 were considered statistically significant.

## Results

*Escherichia coli* O157:H7 strains were detected at varying frequencies in imported beef, sheep and chicken meat. The O157:H7 strain was most prevalent in chicken (6.07%) and beef (5.90%), while in sheep (2.00%), with a significant difference (P < 0.05; Table 1).

**Table 1:** Prevalence of *Escherichia coli* O157:H7 (as inspected by the Saudi Food and Drug Authority) in imported beef, chicken and sheep meat samples in Saudi Arabia in 2017.

Product	Samples	Total	Contaminated	Prevalence (%)
Beef	All samples	562	33	5.90
	Australia	8	0	0.00
	Brazil	91	2	2.20
	Jordan	6	0	0.00
	New Zealand	2	0	0.00
	UAE	15	1	6.70
	Philippines	4	0	0.00
	Spain	1	0	0.00
	India	428	29	6.80
Chicken	All samples	280	17	6.07
	Brazil	230	16	6.96
	Jordan	20	0	0.00
	India	1	0	0.00
	Tunisia	1	0	0.00
	Ukraine	28	1	3.57
Sheep meat	All samples	50	1	2.00
	Australia	1	0	0.00
	New Zealand	2	0	0.00
	India	47	1	2.13

Regarding chicken, the greatest proportion of samples contaminated with *E. coli* O157:H7 was imported from Brazil (6.96%), followed by Ukraine (3.57%), while no contaminated samples were imported from Jordan, India, or Tunisia, with a significant difference (P < 0.05). Regarding beef, the greatest proportion of contaminated samples was imported from India (6.80%), followed by Brazil (2.20%). Finally, all sheep meat samples contaminated with *E. coli* O157:H7 were imported from India (2.1%; Table 1).

The highest frequency of *E. coli* O157:H7 contamination was found in products imported from Indian companies (30 of 476 samples: eight from company A, five from company B, four from company C, three from company D, two from company E, two from company F and six from other companies; Table 2, Supplementary Table 1, 2 and 3). More beef than sheep meat samples imported from India were screened given the high demand for the former in SA in 2017^13^. Therefore, the prevalence of *E. coli* O157:H7 in beef samples was higher than in sheep meat samples (6.80% and 2.13%, respectively). Products imported from Brazilian companies were also frequently contaminated (18 of 321 samples: four from company G, two from company H, two from company I, two from company K and eight from other companies; Table 2, Supplementary Table 1, 2 and 3). In this case, however, the prevalence of *E. coli* O157:H7 in chicken samples was higher than in beef samples (6.96% and 2.20%, respectively). To ensure anonymity, the companies’ names have been replaced with letters A to K.

**Table 2:** Sources of contaminated samples classified by countries and companies.

	Company codes	Number
India	A	8
	B	5
	C	4
	D	3
	E	2
	F	2
	Other *	6
	Total	30
Brazil	G	4
	H	2
	I	2
	K	2
	Other *	8
	Total	18

*Less than two contaminated samples by company.

## Discussion

Contaminated raw meat is the source of 90% of foodborne infections^17^. Thirty-one pathogens, including *E. coli* O157:H7, were responsible for 10 million annual episodes of foodborne illnesses in the United States^4^. In the present study, samples of imported raw meat were obtained from imported meats in the ports of SA, and the prevalence of *E. coli* O157:H7 in these samples was confirmed (Table 1). Meat products imported from India and Brazil were the most frequently contaminated (Table 1).

The prevalence of *E. coli* O157:H7 was the highest in raw meat products imported from India, posing a threat to public health in the Kingdom of Saudi Arabia (Table 2). According to Shinde et al. (2020), *E. coli* O157:H7 was frequently isolated from healthy Indian cattle on both organised and non-organised farms in and around the Pune District in India during 2015. This can be explained by the fact that new generations of cattle may carry the pathogen but may not present any symptoms, thus appearing as heathy livestock; however, the consumption of meat from such asymptomatic carriers of *E. coli* O157:H7 may affect humans, representing a severe public health concern. Furthermore, subsequent studies in the same region revealed the presence of *E. coli* O157:H7 isolates resistant to a number of common antibiotics used for livestock animals against this pathogen, including cefotaxime, streptomycin, penicillin G, kanamycin, ampicillin, tetracycline, gentamycin and piperacillin. These findings, in addition to our results, emphasise the need for the further of assessment of imported meat, specifically from India, to ensure public health safety. In another recent study in China, clinical isolates of *E. coli* exhibited high resistance to conventional antibiotics for livestock, including sulfamethoxazole, trimethoprim/sulfamethoxazole, tetracycline, nalidixic acid and ampicillin^18^.

**Figure 1:**
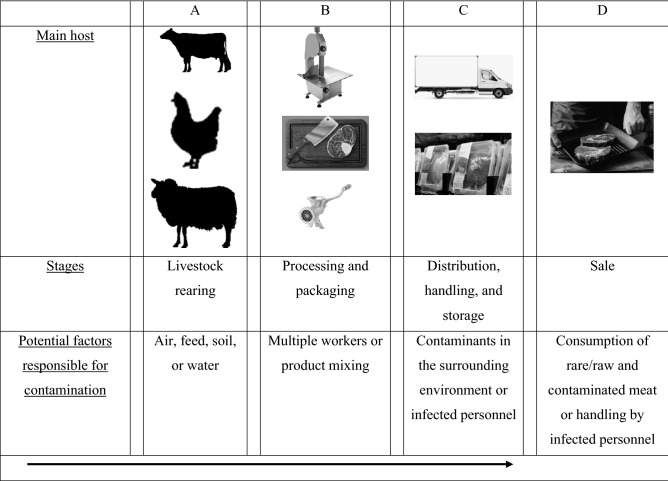
Schematic showing the meat production steps from livestock rearing on farms until sale (**A**,**B**,**C** and **D**). Potential factors responsible for the contamination of meat are illustrated. The arrow indicates the direction of steps from the start to end.

Amongst samples of meat imported from Brazil, *E. coli* O157:H7 was detected at different frequencies in products from several companies (Table 2). The prevalence of *E. coli* O157:H7 in samples from only specific companies (G, H, I, J, K and others), but not others, indicates internal contamination through air during rearing at the livestock farms^19^, slaughter^20^, or processing^5^ (Fig. 1). According to Santos et al. (2018), the prevalence of STEC in Brazilian food products was approximately 9.50%, which was primarily attributed to the development of multi-resistance to antibiotics in these strains. Notably, Brazil is the second largest exporter and the third major producer of beef worldwide^1^.

The detection of *E. coli* O157:H7 in samples of meat imported from one company each in Ukraine and UAE also indicates unhygienic handling that led to contamination (Table 2), highlighting the need for the revision of processing and packaging steps in these regions^5^.

Of note, the present report only includes results from products that have been undergone *E. coli* O157:H7 testing from the port of SA. Many shipments may have been excluded from the examination for approval and owners may have only been asked to produce a list of essential documents^13^. In addition, to import food products into SA, the SFDA mandates a registration certificate authorised by the Saudi health ministry, an industry certificate authorised by the commerce ministry and a quality certificate (e.g. International Organization for Standardization 9001 or 22000, Good Manufacturing Practice and Hazard Analysis Critical Control Point)^13^. Therefore, to ensure public safety, the SFDA has announced a list of countries from where the import of food into SA is prohibited (available at https://www.sfda.gov.sa/en/list_countries).

## Summery

The presence of *E. coli* O157:H7 in samples of imported raw meat highlights the need for more regular surveillance at the borders of SA before the products are made available on the market for consumption by the public. Our results underscore the necessity of more stringent control protocols for the approval of imported food products, particularly from India and Brazil, which are the major suppliers of meat to SA. Moreover, the detected *E. coli* O157:H7 isolates should be tested against antibiotics that are commonly used to treat livestock. For the future investigations and as an alternative method, we suggest tracking different sources of *E. coli* O157 contaminations by clade typing^10^.

## Supplementary Information

Supplementary Information 1.

Supplementary Information 2.

Supplementary Information 3.

Supplementary Information 4.

Supplementary Information 5.

## Data Availability

The All data analysed are reported in this manuscript, and specific reference numbers of samples at SFDA database are listed in supplementary 1, 2 and 3. There is no other data to be provided.

## Abbreviations

SA: Saudi Arabia
SFDA: Saudi Food and Drug Authority
HUS: Haemolytic–uremic syndrome
STEC: Shigatoxigenic *E. coli*
VTEC: Verotoxigenic *E. coli*
EHEC: Enterohaemorrhagic *E. coli*
GCC: Gulf Cooperation Council
GSO: GCC Standardization Organisation
mTSB: Modified Tryptone Soya Broth
ISO: International Organization for Standardization
CT-SMAC: Cefixime tellurite sorbitol MacConkey agar
dH_2_O: Distilled water
RT-PCR: Real-time polymerase chain reaction
SDS: Sequence detection system
LIMS: Laboratory information management system
