# Supplementary material for: Detection of Escherichia coli O157:H7 in imported meat products from Saudi Arabian ports in 2017
Source: Sci Rep. 2023 Mar 14;13:4222. doi: 10.1038/s41598-023-30486-2 (PMC10015049; doi:10.1038/s41598-023-30486-2)
Supplement: Supplementary file 4 — Supplementary Information 4. [file 41598_2023_30486_MOESM4_ESM.docx]

Supplementary Table 4. Summarizing sources of tested samples against *E. coli* O157:H7 in this study

| No. | Code used in this study | Manufacturer Country | Beef/ (+) | Chicken/ (+) | Sheep/ (+) | Total/ (+) |
| --- | --- | --- | --- | --- | --- | --- |
| 1 | Company L | Australia | 1 (0) | Non | Non | 1 (0) |
| 2 | Company M | Australia | 2 (0) | Non | Non | 2 (0) |
| 3 | Company N | Brazil | 25 (1) | Non | Non | 25 (1) |
| 4 | Company O | Brazil | 2 (0) | Non | Non | 2 (0) |
| 5 | Company P | Brazil | 1 (0) | Non | Non | 1 (0) |
| 6 | Company Q | Brazil | 2 (0) | Non | Non | 2 (0) |
| 7 | Company R | Brazil | 1 (0) | Non | Non | 1 (0) |
| 8 | Company S | Brazil | 7 (0) | Non | Non | 7 (0) |
| 9 | Company T | Brazil | 1 (0) | Non | Non | 1 (0) |
| 10 | Company U | Brazil | 1 (0) | Non | Non | 1 (0) |
| 11 | Company V | Brazil | 3 (0) | Non | Non | 3 (0) |
| 12 | Company W | Brazil | 2 (0) | Non | Non | 2 (0) |
| 13 | Company X | Brazil | 1 (0) | Non | Non | 1 (0) |
| 14 | Company Y | Brazil | 2 (0) | Non | Non | 2 (0) |
| 15 | Company Z | Brazil | 1 (0) | Non | Non | 1 (0) |
| 16 | Company AA | Brazil | 1 (0) | Non | Non | 1 (0) |
| 17 | Company AB | Brazil | 2 (0) | Non | Non | 2 (0) |
| 18 | Company AC | Brazil | 2 (0) | 6 (1) | Non | 8 (1) |
| **19** | **Company G** | **Brazil** | **2 (0)** | **59 (4)** | **Non** | **61 (4)** |
| 20 | Company AD | Brazil | 1 (0) | Non | Non | 1 (0) |
| 21 | Company AE | New Zealand | 2 (0) | Non | Non | 2 (0) |
| 22 | Company AF | UAE | 2 (0) | Non | Non | 2 (0) |
| 23 | Company AG | UAE | 1 (0) | Non | Non | 1 (0) |
| 24 | Company AH ǂ | UAE | 12 (0) | Non | Non | 12 (0) |
| 25 | Company AI ǂ | India | 7 (0) | Non | 45 (1) | 52 (1) |
| 26 | Company AJ ǂ | Brazil | Non | 2 (0) | Non | 2 (0) |
| 27 | Company AK | Philippines | 4 (0) | Non | Non | 4 (0) |
| 28 | Company AL | India | 2 (1) | Non | Non | 2 (1) |
| **29** | **Company B** | **India** | **108 (5)** | **Non** | **Non** | **108 (5)** |
| **30** | **Company D** | **India** | **34 (4)** | **Non** | **Non** | **34 (4)** |
| **31** | **Company F** | **India** | **8 (2)** | **Non** | **Non** | **8 (2)** |
| 32 | Company AM | India | 2 (1) | Non | Non | 2 (1) |
| **33** | **Company C** | **India** | **17 (3)** | **Non** | **Non** | **17 (3)** |
| 34 | Company AN | India | 19 (1) | Non | Non | 19 (1) |
| 35 | Company AO | India | 10 (0) | Non | Non | 10 (0) |
| 36 | Company AP | India | 1 (0) | Non | Non | 1 (0) |
| 37 | Company AQ | India | 32 (0) | Non | Non | 32 (0) |
| **38** | **Company E** | **India** | **103 (2)** | **Non** | **Non** | **103 (2)** |
| **39** | **Company A** | **India** | **21 (8)** | **Non** | **Non** | **21 (8)** |
| 40 | Company AR | India | 3 (0) | Non | Non | 3 (0) |
| 41 | Company AS | India | 1 (0) | Non | Non | 1 (0) |
| 42 | Company AT | India | 6 (0) | Non | Non | 6 (0) |
| 43 | Company AU ₸ | India | 6 (0) | Non | Non | 6 (0) |
| 44 | Company AV ₸ | Brazil | Non | 1 (0) | Non | 1 (0) |
| 45 | Company AW | India | 1 (0) | Non | Non | 1 (0) |
| 46 | Company AX | India | 1 (0) | Non | Non | 1 (0) |
| **47** | **Company I** | **Brazil** | **Non** | **18 (2)** | **Non** | **18 (2)** |
| 48 | Company AY | Brazil | Non | 14 (1) | Non | 14 (1) |
| 49 | Company K | Brazil | 1 (1) | 7 (1) | Non | 8 (2) |
| 50 | Company AZ | Brazil | Non | 4 (1) | Non | 4 (1) |
| 51 | Company BA | Brazil | Non | 2 (1) | Non | 2 (1) |
| 52 | Company BB | Brazil | Non | 3 (1) | Non | 3 (1) |
| 53 | Company BC | Brazil | Non | 2 (1) | Non | 2 (1) |
| 54 | Company BD | Brazil | Non | 3 (1) | Non | 3 (1) |
| 55 | Company BE | Brazil | Non | 4 (0) | Non | 4 (0) |
| 56 | Company BF | Brazil | Non | 11 (0) | Non | 11 (0) |
| 57 | Company BG | Brazil | Non | 1 (0) | Non | 1 (0) |
| 58 | Company BH | Brazil | Non | 1 (0) | Non | 1 (0) |
| 69 | Company BI | Brazil | Non | 3 (0) | Non | 3 (0) |
| 70 | Company H | Brazil | Non | 33 (0) | Non | 33 (0) |
| 71 | Company BJ | Brazil | Non | 4 (0) | Non | 4 (0) |
| 72 | Company BK | Brazil | Non | 2 (0) | Non | 2 (0) |
| 73 | Company BL | Brazil | Non | 4 (0) | Non | 4 (0) |
| 74 | Company BM | Brazil | Non | 1 (0) | Non | 1 (0) |
| 75 | Company BN | Brazil | Non | 9 (0) | Non | 9 (0) |
| 76 | Company BO | Brazil | Non | 5 (0) | Non | 5 (0) |
| 77 | Company BP | Brazil | Non | 1 (0) | Non | 1 (0) |
| 78 | Company BQ | Brazil | Non | 2 (0) | Non | 2 (0) |
| 79 | Company BR | Brazil | Non | 1 (0) | Non | 1 (0) |
| 80 | Company BS | Brazil | Non | 2 (0) | Non | 2 (0) |
| 81 | Company BT | Brazil | Non | 3 (0) | Non | 3 (0) |
| 82 | Company BU | Brazil | Non | 2 (0) | Non | 2 (0) |
| 83 | Company BV | Brazil | Non | 1 (0) | Non | 1 (0) |
| 84 | Company BW | Brazil | Non | 1 (0) | Non | 1 (0) |
| 85 | Company BX | Brazil | Non | 1 (0) | Non | 1 (0) |
| 86 | Company BY | Brazil | Non | 2 (0) | Non | 2 (0) |
| 87 | Company BZ | Jordan | Non | 8 (0) | Non | 8 (0) |
| 88 | Company CA | Tunisia | Non | 1 (0) | Non | 1 (0) |
| 89 | Company CB | Ukraine | Non | 23 (1) | Non | 23 (1) |
| 90 | Company CC | Ukraine | Non | 1 (0) | Non | 1 (0) |
| 91 | Company CD | Ukraine | Non | 2 (0) | Non | 2 (0) |
| 92 | Company CE | Australia | Non | Non | 1 (0) | 1 (0) |
| 93 | Company CF | New Zealand | Non | Non | 1 (0) | 1 (0) |
| 94 | Company CG | India | Non | Non | 1 (0) | 1 (0) |
| 95 | Company CH | India | 1 (0) | Non | Non | 1 (0) |
| 96 | US | Australia | 5 (0) | Non | Non | 5 (0) |
| 97 | US | Brazil | 32 (0) | 4 (0) | Non | 36 (0) |
| 98 | US | Jordan | 6 (0) | 12 (0) | Non | 18 (0) |
| 99 | US | New Zealand | Non | Non | 1 (0) | 1 (0) |
| 100 | US | UAE | Non | Non | Non | Non |
| 101 | US | Philippines | Non | Non | Non | Non |
| 102 | US | Spain | 1 (0) | Non | Non | 1 (0) |
| 103 | US | India | 46 (2) | 1 (0) | 1 (0) | 48 (2) |
| 104 | US | Tunisia | Non | Non | Non | Non |
| 105 | US | Ukraine | Non | 2 (0) | Non | 2 (0) |
| Total | | | 555 (31) | 269 (15) | 50 (1) | 874 (47) |

UAE: United Arab Emirates.

ǂ / ₸: Factories under same brand name were exporting meat to Saudi Arabia from different country.

Data in bold are discussed in this study.

US: Unknown Sources
