# Supplementary material for: Detection of Escherichia coli O157:H7 in imported meat products from Saudi Arabian ports in 2017
Source: Sci Rep. 2023 Mar 14;13:4222. doi: 10.1038/s41598-023-30486-2 (PMC10015049; doi:10.1038/s41598-023-30486-2)
Supplement: Supplementary file 5 — Supplementary Information 5. [file 41598_2023_30486_MOESM5_ESM.docx]

Table S.1. Thermal-cycling conditions of the RT-PCR amplification used in this study

| Step | Enzyme activation | PCR | |
| --- | --- | --- | --- |
|  | HOLD | Cycle (40 cycles) | |
|  |  | Denature | Anneal/extend |
| Temperature | 95°C | 95°C | 60°C |
| Time | 2 min | 3 sec | 30 sec |
